# Nonleukemic Granulocytic Sarcoma of Knee: A Case Report

**DOI:** 10.1155/2010/235295

**Published:** 2010-12-14

**Authors:** Ibrahim Elghissassi, Hanane Inrhaoun, Hind Mrabti, Hassan Errihani

**Affiliations:** Department of Medical Oncology, National Institute of Oncology, Allal El-Fassi Street, Rabat, Morocco

## Abstract

Granulocytic sarcoma (GS) is a rare extramedullary tumor composed of immature myeloid cells. It is usually associated with leukemia or other myeloproliferative disorders. It occurs very rarely without overt hematologic diseases. A 19-year-old man presented with left knee mass. Biopsy with pathological analysis showed lymphoma aspect. Immunostains yielded the diagnosis of GS with myeloperoxidase and CD43 positivity. There was no systemic manifestation of leukemia, and bone marrow biopsiy was negative for neoplastic infiltration. Chemotherapy by CHOP was efficient, and the patient remaind alive and healthy 40 months after the end of treatment. The case is discussed in the framework of the existing literature about the diagnosis, treatment, and prognosis of this very rare condition.

## 1. Background

Granulocytic sarcoma (GS) is a rare localized tumor composed of immature granulocytic precursors. These neoplasms are known as monocytic sarcoma and chloroma which refers to the green coloration of some of these tumors on gross examination [1]. It is usually associated with leukemia or other myeloproliferative disorders [2]. However, it occurs very rarely without hematologic diseases [3], and the diagnosis is difficult in such cases. 

The most common sites of involvement are the skin, lymph nodes, soft tissues, and bone, but the tumor can occur virtually anywhere [2–6]. Although nonleukemic GS may be found in any location, primary occurrence in the knee is exceptional. Here we report a very rare case of GS of the knee but no bone marrow involvement at presentation which was accurately diagnosed and has been followed up for a long period.

## 2. Case Report

**Figure 1:**
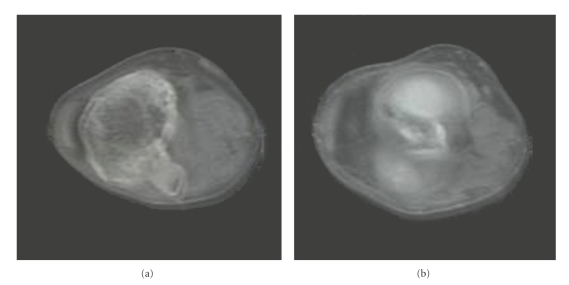
A large mass of anterior left knee involving bone and soft tissue.

A 19-year-old Berber Moroccan man began experiencing pain in his left knee. His medical history was otherwise unremarkable. Physical examination revealed no abnormality except for an 8 cm, mobile mass of left knee with no involvement of overlying skin. CTscan revealed a large mass of anterior left knee involving bone and soft tissue suspicious of sarcoma (Figure 1). A radionuclide bone scan showed a single area of uptake of radiotracer in the left knee.

Biopsy showed a green colour of the tumour, and pathological analysis revealed diffuse proliferation of round cells with medium-sized or large nuclei lymphoma aspect. Immunostains showed myeloperoxidase and CD43 positivity and leukocyte common antigen (LCA) and CD 3, 5, 56 negativity indicating a diagnosis of a well-differentiated granulocytic sarcoma. There was no systemic manifestation of leukemia. The blood cell count, thoracoabdominal CTscan, and bone marrow histology were normal. 

The patient received 6 cycles of chemotherapy by CHOP (consisting of 750 mg/m2 cyclophosphamide intravenously injected on day 1, 50 mg/m2 doxorubicin injected intravenously, on day 1; 1.4 mg/m2 vincristine intravenously injected on day 1, and 100 mg prednisolone orally administrated on days 1–5 repeated every three weeks). The patient responded well to the treatment, and complete remission was achieved. He remained alive and healthy 40 months after the end of treatment. 

## 3. Discussion

GS is a rare extramedullary tumor that consists of immature granulocytic cells. this tumor was first described in 1811 and originally called chloroma by King in 1853 [7]. The term “chloroma” is derived from the Greek word chloros (green). The estimated incidence is about 0,7 per million in children and 2 per million in adults [8]. There is no sex predilection, with a mean age of 48 years (range: 2–81) [1]. GS occurs most commonly in bone, periosteum, soft tissue, lymph nodes, and skin, although it can occur anywhere throughout the body [9]. 

GS usually presents concomitantly with or after the onset of acute myelogenous leukemia, blastic phase of chronic myelogenous leukemia, or myelodysplastic syndrome [9]. However, it is seen very rarely without bone marrow disease as in our case and symptoms are secondary to the mass effect of the tumor. To the best of our knowledge, there has been only one case report of nonleukemic GS of the knee in the medical literature [10]. 

Macroscopically, GS is usually green in appearance. The green color, which is due to the presence of myeloperoxidase (MPO) in the leukemia cells, is not present in all tumors of this type [11]. According to the World Health Organization classification, the tumor is histologically classified into 3 types: well-differentiated, poorly differentiated, and blastic [12, 13]. The diagnosis is often difficult in GS cases when the myeloblastic cells are poorly differentiated and the tumor lacks the characteristic green color. A large proportion (75%–86%) of GS in nonleukemic patients is initially misdiagnosed and frequently mistaken for non-Hodgkin lymphoma, small round cell tumor (neuroblastoma, rhabdomyosarcoma, Ewing sarcoma/PNET, and medulloblastoma), and undifferentiated carcinoma [8]. In such isolated presentation, early diagnosis is based on immunohistochemical analysis, especially staining of MPO and CD43. Traweek et al. suggested that an immunohistochemical panel including CD20, CD43, CD68, and MPO can successfully identify 96% of extramedullary myeloid cell tumors via paraffin sections [13].

The rarity of cases of nonleukemic GS has limited the availability of prospective studies on the effectiveness of various treatment strategies. Although no standard treatment protocol has been established, several previous reports suggested that local treatments such as surgical excision and/or radiation therapy alone would not be sufficient for the treatment of nonleukemic GS [1, 2, 14] and early aggressive chemotherapy may represent the best chance for remission [14–17]. Our patient received 6 cycles of CHOP-regimen because he refused bone marrow transplantation. 

The long-term prognosis of nonleukemic GS remains poor [15], and the majority of these patients die of leukemia within an average of 16.5 months after diagnosis [9]. Uncommonly, however, cases of no progression have also been reported [18, 19]. In our case, the patient has remained free of disease for 40 months.

GS is rare, and clinical diagnosis is especially difficult without bone marrow involvement. Immunohistochemical studies using an appropriate panel of markers are extremely helpful to make a correct diagnosis. The strategy for the management of nonleukemic GS should be individualized. Aggressive chemotherapy is beneficial and may induce complete remission. Early diagnosis and strict followup of these patients are essential for a better outcome in GS.

##  Consent

Written informed consent was obtained from the patient's father for publication of this case report. A copy of the written consent is available for review with the Editor-in-Chief of this journal.

##  Conflict of Interests

The authors declare that they have no conflict of interests.

##  Authors' Contributions

I. Elghissassi conceived the case report, analyzed and interpreted the patient data regarding the haematological disease H. Innrhaoun analyzed and interpreted the patient data and was a major contributor in the literature review H. Mrabti analyzed and interpreted the patient data and was a major contributor in the literature review H. Errihani contributed to revision, supervision and approval of the work. All authors read and approved the final manuscript.

## Abbreviations

GS: Granulocytic sarcoma
CD: Cluster of differentiation
CHOP: Cyclophosphamide, doxorubicin, vincristine and prednisolone
LCA: Leukocyte common antigen
MPO: Myeloperoxidase
PNET: Primitive neuroectodermal tumor.
